# Les communications interauriculaires chez l’enfant: diagnostic et prise en charge à propos de 49 cas aux CHU pédiatriques de Dakar

**DOI:** 10.11604/pamj.2018.30.245.14556

**Published:** 2018-08-02

**Authors:** Idrissa Basse, Amadou Lamine Fall, Ndiogou Seck, Djiril Boiro, Aïssatou Ba, Ndiémé Ndiaye Diawara, Fatou Niang, Dina Cyrienne Obambi Diop, Aliou Abdoulaye Ndongo, Abou Ba, Lamine Thiam, Ndéye Rama Diagne/Guéye, Mouhamadou Ndiaye

**Affiliations:** 1 Service de Pédiatrie, Hôpital pour Enfants de Diamniadio, Université de Thiès, Dakar, Sénégal; 2 Hôpital d’Enfants Albert Royer, Université Cheikh Anta Diop (UCAD) de Dakar, Sénégal; 3 Service de Pédiatrie, Centre Hospitalier Régional de Saint Louis, Université de Saint Louis, Dakar, Sénégal; 4 Service de Pédiatrie, Centre Hospitalier National Abass Ndao, Université CAD de Dakar, Sénégal; 5 Service de Pédiatrie, Hôpital Aristide Le Dantec, Université Cheikh Anta Diop de Dakar, Sénégal; 6 Service de Pédiatrie, Hôpital de la paix de Ziguinchor, Université de Ziguinchor, Dakar, Sénégal; 7 Service de Chirurgie Thoracique et Cardio-vasculaire, Hôpital de Fann, UCAD, Dakar, Sénégal

**Keywords:** Cardiopathie, communication interauriculaire, échographie, Heart disease, interauricular communication, ultrasound

## Abstract

La communication interauriculaire (CIA) constitue la seconde cardiopathie congénitale la plus importante chez l'enfant, elle représente 6 à 8% des malformations congénitales cardiaques de l'enfant. Toutefois, de nombreuses questions demeurent ouvertes concernant cette pathologie. Ainsi notre objectif était de rapporter la prévalence hospitalière des CIA mais surtout d'en décrire les aspects cliniques, paracliniques et thérapeutiques. Pour y parvenir nous avons réalisé une étude rétrospective descriptive. Les données ont été recueillies grâce à un questionnaire puis saisies et analysées sur Sphinx (V5). La prévalence hospitalière était de 2 pour 1000. L'âge moyen était de 37mois et le sex-ratio de 0,75. Il n'y a eu aucun cas diagnostiqué en anténatal. Une consanguinité parentale était retrouvée dans 30% des cas. Les infections respiratoires étaient présentes dans 24% des cas. Une cardiomégalie était retrouvée dans 35 cas avec une hyper vascularisation pour 63%. L'échographie notait une prédominance du type ostium secundum; la CIA était large dans 63% des cas. La sténose pulmonaire représentait l'atteinte cardiaque associée la plus importante. L'HTAP était présente dans 63% des cas. Les diurétiques étaient très utilisés et seuls 7 enfants avaient bénéficié d'une cure chirurgicale. L'évolution était favorable chez 39 patients soit 79%. Le diagnostic précoce des CIA doit être amélioré ainsi que la prise en charge chirurgicale des formes avec retentissement du nourrisson.

## Abstract (en)

Interauricular communication (IAC) is the second most important congenital heart disease in children. It accounts for 6-8% of congenital cardiac malformations in children. However, many questions are still open about this pathology. Thus, this study aims to report the prevalence rate of IAC in hospital but especially to describe its clinical, paraclinical and therapeutic aspects. For this purpose, we conducted a retrospective descriptive study. Data were collected by means of a questionnaire and then entered and analyzed in Sphinx (V5). The prevalence in Hospital was 2x1000. The average age of patients was 37 months, the sex-ratio was 0,75. No prenatal diagnosis was made. Parental consanguinity was detected in 30% of cases. Respiratory infections were found in 24% of cases. Cardiomegaly was found in 35 cases with hypervascularization in 63% of cases. Ultrasound showed a predominance of ostium secundum; wide IAC was found in 63% of cases. Pulmonary stenosis was the most important associated heart attack. Pulmonary arterial hypertension was found in 63% of the cases. Treatment was based on diuretics, which were largely used and only 7 children underwent surgery. Evolution was favorable in 39 patients reflecting a rate of 79%. Early diagnosis of IAC should be improved as well as surgical treatment of IAC affecting infants.

## Introduction

La communication interauriculaire (CIA) est une pathologie cardiaque congénitale définie par la persistance d'une communication entre les deux oreillettes, secondaire à un défaut de cloisonnement septal lors de l'embryogénèse créant ainsi un shunt entre ces deux cavités. On décrit habituellement quatre types anatomiques de CIA selon la localisation du défect pariétal (ostium primum, ostium secundum, sinus venosus, sinus coronaire) [1]. En effet, la connaissance de cette malformation et de ses diverses variétés anatomiques a été révolutionnée par le progrès de l'échocardiographie cardiaque. La CIA touche 1 sur 1000 naissances vivantes et représente 6 à 8% des malformations congénitales cardiaques de l'enfant. La prévalence dans la population générale est estimée à 1/25.000 [2]. Très peu étudiée en Afrique et au Sénégal en particulier, de nombreuses questions demeurent ouvertes concernant cette pathologie. Faut-il traiter toutes les CIA diagnostiquées? Si oui, quel serait le moment idéal pour intervenir? Et quelle méthode privilégier? Un traitement médical d'appoint ou une correction chirurgicale ou instrumentale de la communication? Ainsi nous avions entrepris ce travail dont l'objectif était d'apprécier les aspects du diagnostic et du traitement des CIA afin de mieux comprendre certaines questions suscitées.

## Méthodes

L'étude a été menée à Dakar au Centre Hospitalier National d'Enfants Albert Royer (CHNEAR) et au service de Chirurgie Thoracique et Cardiovasculaire du CHUN de Fann (CTCV). Le CHNEAR est une structure de référence d'une capacité de 170 lits qui reçoit les enfants de 0 à 15 ans, il dispose d'une unité de cardiologie où sont menées des activités de suivi des enfants souffrant de maladies cardiaques. La prise en charge chirurgicale de ces enfants s'il y a lieu se fait au service de CTCV du CHU de Fann. Il s'agissait d'une étude rétrospective, descriptive qui s'est intéressée aux aspects cliniques, paracliniques et à la prise en charge des enfants présentant une CIA durant la période d'Octobre 2010 à Septembre 2015, soit une période de 5 ans. Etaient inclus, tous les enfants chez qui une CIA a été diagnostiquée à l'échographie. L'appareil utilisé avait comme caractéristiques: PHILIPS SONOS 7500, année 2003, sondes S4 et S8. Les données ont été recueillies à partir des dossiers médicaux des patients et des registres d'hospitalisation, en se basant sur une fiche de recueil pré-établie. Les paramètres étudiés étaient: les données sociodémographiques: âge, sexe, origine géographique, niveau socio-économique; Les données cliniques: motifs d'admission, données anthropométriques, signes fonctionnels et d'examen physique les données paracliniques: Index cardiothoracique, signes radiologiques pulmonaires, signes électriques (Hypertrophie auriculaire ou ventriculaire, troubles du rythme ou de la conduction), données échographiques (Situs, type CIA, taille CIA, retentissement cavitaire, pressions pulmonaires, cardiopathie associée); Les données concernant la prise en charge (traitement d'urgence, en cours d'hospitalisation, moyen chirurgical ou interventionnel); Les données évolutives (complications aiguës et létalité).

Les données ont été saisies et analysées à l'aide du logiciel sphinx (v5) et Microsoft office Excel 2010 et 2013 sous Windows 7 et 8. Les variables quantitatives ont été étudiées en déterminant le maximum, le minimum, la moyenne et l'écart type alors que les variables qualitatives ont été analysées en déterminant la fréquence et le pourcentage. L'analyse de nos données a été faite grâce à la méthode de chi carré (chi ^2^) et Mc Nemar, et un p≤0,05 est considéré comme statistiquement significatif.

## Résultats

### Données épidémiologiques et sociodémographiques

Durant la période d'étude de 5 ans, 49 cas ont été colligés soit une prévalence hospitalière de 2,5 pour 1000 et une prévalence par rapport aux cardiopathies congénitales de 8%. Une prédominance féminine était notée avec un sex-ratio de 0,75. L'âge moyen de nos patients au moment de leur hospitalisation était de 37 mois avec un âge médian 12,5 mois pour des extrêmes de 1 mois et 15 ans ; et la tranche d'âge 1-30 mois était plus représentée. Soixante-deux pour cent (62%) des patients (n=29) provenaient de la banlieue dakaroise, 23% de Dakar centre (n=11), 15% venaient des autres régions (n=7). Le niveau socio-économique était faible pour la plupart avec 83%.

### Données cliniques

En anténatal, la majorité des mères avaient effectué 3 consultations prénatales ou plus (97%). Aucun cas de CIA n'a été diagnostiqué avant la naissance. Une consanguinité était notée chez 51% des enfants, au second degré dans 73% des cas, premier degré dans 7% des cas (1 enfant) et au troisième degré dans 20% des cas (3 enfants). En post natal une mauvaise croissance pondérale était notée dans 31% des cas et 84% avaient un bon développement psychomoteur. La vaccination était à jour chez 96% de nos patients (n=47). Les infections respiratoires constituaient les antécédents médicaux les plus fréquents avec 50% (n=10) suivies de l'insuffisance cardiaque avec 20% (n=4). Une trisomie 21 était associée dans 10% (n=5) des cas. La cardiopathie était connue antérieurement à l'hospitalisation dans 49% des cas. Les patients étaient admis pour prise en charge de la CIA dans 59% (n=29) suivi des infections respiratoires 24% (n=12), l'insuffisance cardiaque 20% (n=10) et la détresse respiratoire 20% (n=10), (Tableau 1). La dyspnée est la manifestation clinique la plus fréquente avec 36% soit 18 cas suivi de la toux avec 20% soit 10 cas et de la fièvre avec 12,2% soit 6 cas (Tableau 2).

**Tableau 1:** Répartition des patients selon les motifs d’admission

Motifs d’admission	Effectif	Pourcentage (%)
Prise en charge CIA	29	59
Infections pulmonaires	12	24
Insuffisance cardiaque	10	20
Difficultés respiratoires	10	20
Prise en charge cardiopathie congénitale	09	16
Syndrome infectieux	05	8,8
Prise en charge diarrhée	02	3,5

**Tableau 2:** Répartition des patients selon les manifestations cliniques

cliniques à l’admission	Effectifs	Pourcentage (%)
Fonctionnels		
Dyspnee	18	37
Toux	10	20,41
Autres	2	4,08
Généraux		
Fièvre	6	12,24
Cyanose	1	2,04
Retard ponderal	1	2,04
Physiques		
Detresse respiratoire	2	4,08
Souffle mesocardiaque	1	2,04

Autres = douleurs abdominales et diarrhée

### Données paracliniques

**L'échographie cardiaque:** a révélé 18 cas de CIA isolées et 31 cas de CIA associées à d'autres malformations cardiaques. Le type anatomique le plus fréquemment retrouvé était le type ostium secundum dans 70% des cas. La sténose pulmonaire représentait l'atteinte cardiaque associée la plus fréquente avec 14 cas soit 45% suivie de la communication inter ventriculaire (CIV) qui est à 32% soit 10 cas, ensuite vient la persistance de canal artériel (PCA) à 19% soit 6 cas et 2 cas d'insuffisance mitrale soit 6% (Tableau 3). Pour les CIA isolées, 50% (n=9) étaient de taille large (supérieur à 5mm), la taille était petite (inférieure à 3mm) dans 33% (n=6) et 17% (n=3) étaient de taille moyenne (3 à 5mm). Dans 50% des cas de CIA isolées (n=9) les cavités droites étaient dilatées avec une HTAP dans 61% des cas (n=11). Dans les CIA avec d'autres malformations associées, cinquante-huit pour cent (58%) (n=18) étaient de taille large (supérieur à 6mm), la taille était moyenne (3 à 5mm) dans 29% (n=9), 13%(n=3) étaient de petite taille (inferieure à 3mm); les cavités droites étaient dilatées dans 67% soit 18 cas avec une HTAP dans 70% des cas.

**Tableau 3:** Répartition des patients selon les cardiopathies associées à la CIA

Cardiopathies	Effectifs	Pourcentage
Sténose pulmonaire	14	45%
CIV	10	32%
PCA	6	19%
IM	2	6%
Autres	4	16%

Autres = canal atrio ventriculaire et atrésie tricuspidienne

**La radiographie du thorax:** dans les CIA isolées une cardiomégalie était retrouvée dans 67% soit chez 12 enfants avec un ICT moyen de 0,63; une hypervascularisation pulmonaire était notée dans 61% des cas (n=11) avec 2 cas de pneumonies et 1 cas d'œdème aigu du poumon. Dans les CIA associées à d'autres malformations une cardiomégalie était présente dans 74% des cas (n=23), une hypervascularisation dans 61% des cas, une hypo vascularisation dans 3%; Une bronchiolite était associée dans 60% des cas et une pneumonie dans 40%.

**L'électrocardiogramme:** pour les cas de CIA isolées, l'ECG a été réalisée chez 7 patients; le rythme était sinusal et régulier dans 90% des cas, l'axe QRS était droit pour les 3 cas où il était précisé. Une hypertrophie ventriculaire droite (HVD) était présente dans 43% des cas (n=3). Dans les CIA associées à d'autres malformations, l'ECG était réalisé chez 6 patients; le rythme était sinusal et régulier dans 83% des cas (n=5) avec un axe droit pour 80%; Une HVD était notée chez 3 patients (50%). Sur le plan biologique, l'anémie 63% (n=27) avec un taux d'hémoglobine (HGB) moyen de 10,08g/dl, l'hyperleucocytose 47% (n=20) avec une moyenne de 15000/μl, une thrombocytose 16% (n=7) avec une moyenne de 659 571, étaient les anomalies les plus fréquemment retrouvées.

### Données thérapeutiques

Les traitements prescrits en hospitalisation étaient constitués de diurétiques dans 91% des cas (n=45), d'antibiotiques dans 20 cas soit 40%, d'antalgiques dans 10 cas soit 20%, de bêtamimétiques dans 9 cas soit 18%, d'oxygène dans 6 cas soit 10%, de bétabloquants dans 3 cas et digitaliques dans 3 cas soit 6%. Sept enfants ont bénéficiés de traitement chirurgical curatif soit 14,2%, avec 5 cas de fermeture par Patch et 2 cas de fermeture directe. Pour les atteintes cardiaques associées un cerclage de l'artère pulmonaire a été réalisé dans 3 cas, 3 cures de sténose pulmonaire et une fermeture de PCA. Tous nos patients ont bénéficiés de CEC. L'évolution en cours d'hospitalisation était favorable chez 39 patients soit 79%. Cependant on avait constaté des complications chez 7 patients soit 14% dont les principales étaient marquées par l'insuffisance cardiaque globale et la détresse respiratoire. Trois cas soit 6% de décès ont été notés y compris les 2 cas de la chirurgie. L'analyse a permis de montrer qu'il y'a un lien significatif entre l'âge et la prise en charge chirurgicale, de même qu'entre l'existence d'une insuffisance cardiaque et la chirurgie curative.

### L'évolution

L'évolution en cours d'hospitalisation était favorable chez 39 patients soit 79%. Cependant on avait constaté des complications chez 7 patients soit 14% dont les principales étaient marquées par l'insuffisance cardiaque globale et la détresse respiratoire. Trois cas soit 6% de décès ont été notés dont 2 cas après chirurgie.

## Discussion

Avec l'amélioration des moyens de diagnostic des cardiopathies congénitales, leur épidémiologie se modifie au fil des années. Ainsi d'une prévalence des CIA de 4,7% des cardiopathies congénitales en 2012 [3], nous avons retrouvé une prévalence plus élevée de 8%. Au Congo en 2015, Nika E.R. rapportait une prévalence similaire de l'ensemble des cardiopathies congénitales opérées [4]. En France avec des moyens de diagnostic beaucoup plus performants, cette prévalence est de 10% [5]. Le sex-ratio était de 0,75 dans notre travail, cette excès de filles dans les CIA a été rapporté par Robert Gnansia en France avec cependant un sex-ratio qui restait constant pour les cardiopathies congénitales de façon globale (1,06) [6]. D'autres études ont rapporté cette prédominance féminine [7, 8] par contre ELALJ [9] au Maroc en 2010 retrouvait un sex-ratio de 1,03 des CIA. L'âge moyen de notre population d'étude était de 37 mois avec des extrêmes de 1 mois et 15 ans. Cet âge moyen est variable tel que l'ont montré les études qui ont été faites au Sénégal par WONE [3] où l'âge moyen était de 2 mois et DIOP [10] avec un âge moyen de 11 mois et demi. Cependant, comme l'a montré notre étude où 63% des patients étaient des nourrissons, ces mêmes études [3, 10] ont rapporté des résultats similaires. Au Maroc, Elalj retrouvait un âge moyen de 23 mois [9]. Cependant en France Maingourd et Dupuis rapportaient une découverte de la CIA se faisant à l'âge scolaire, le plus souvent au cours d'une visite systématique [11, 12]. Ceci montre l'importance du diagnostic anténatal qui reste très avancé en occident. Nous n'avons pas eu de cas diagnostiqué avant la naissance comme ce fut le cas lors d'études précédentes menées à Dakar [3, 10]. Les âges des mamans étaient en majorité compris entre 18 et 35 ans dans notre série mais dans la littérature il semble exister une augmentation modérée du risque pour l'ensemble des cardiopathies peu sévères chez les mères de plus de 35 ans [6]. L'origine génétique étant toujours incriminée dans la survenue de cardiopathies congénitales, nous avons constaté dans notre série une consanguinité dans 15% des cas qui était de 2nd degré pour 73%. La consanguinité était plus présente dans le travail de Wone en 2015 avec 30% des cas de cardiopathies congénitales. Comme l'ont rapporté plusieurs études [13, 14], les affections respiratoires restent l'antécédent médical d'hospitalisation le plus fréquent; nous avons fait le même constat avec 50% des cas qui présentait une bronchiolite ou une pneumonie. La CIA est une cardiopathie bien tolérée sur le plan hémodynamique, sa découverte est souvent tardive pouvant se faire après 10 ans [15-17]. Nous avons constaté que chez 25 enfants soit 51% des cas, la CIA a été découverte de façon fortuite. Près de 50% des enfants de la série nous ont été adressés pour le suivi cependant les affections respiratoires (25%) et l'insuffisance cardiaque (20%) sont restées des motifs fréquents d'admission comme ça a été le cas lors d'études précédentes menées au Sénégal et au Maroc [3, 14]. En Europe, avec plus de moyens de diagnostic et de prise en charge, on retrouve rarement des cas de CIA en insuffisance cardiaque [11, 18].

Du point de vue échographique, dans 36,7% des cas, la CIA était isolée et dans 63,3% des cas la CIA était associée à d'autres malformations cardiaques. Ailleurs dans la sous région deux études faites l'une au Maroc et l'autre au Burkina Faso rapportaient des résultats où les cas de CIA isolée étaient plus fréquents [9, 17]. Tous nos cas de CIA isolées étaient de type ostium secundum. Au Sénégal, dans la majorité des études [3, 10, 13] le type ostium secundum était au premier plan. Ainsi, comme rapporté dans la littérature le type anatomique ostium secundum est plus fréquent [1, 15]. Les enfants qui ont une CIA de taille large semblaient plus représentés car ces formes sont très souvent responsables d'une décompensation cardiaque et nécessitent parfois une chirurgie. Les formes de petites tailles et moyennes sont moins représentées car ce sont des formes de diagnostic fortuit ou qui passent inaperçues et ne nécessitent aucun suivie car peuvent se fermer spontanément. Une dilatation cavitaire droite était notée dans 50% des cas. Cependant dans l'étude de Diop en 2007, on retrouvait un retentissement cavitaire droit dans 83,3% [10]. Une HTAP était présente dans 61% des cas, cependant l'étude de DIOP [10] retrouvait une HTAP dans 33,3%. Concernant les malformations cardiaques associées, la sténose pulmonaire était au premier plan dans notre travail (45%), suivie des communications interventriculaires (CIV) et de la persistance du canal artériel (PCA). D'autres études faites au Sénégal montraient le même ordre mais à des fréquences différentes [3, 10, 13]. Au Maroc, dans le travail de l'ELALJ, la PCA était plus fréquemment associée suivie de la CIV [9]. Connue comme étant un bon examen d'orientation aux demandes d'échographies cardiaques, la radiographie est d'un apport non négligeable pour le diagnostic des lésions pulmonaires souvent associées mais également du retentissement de la CIA. La radiographie révèle dans la plupart des cas une cardiomégalie et/ou une infection pulmonaire comme l'ont montré beaucoup d'études sur les CIA ou les cardiopathies congénitales en général [3, 10, 15, 17].

L'ECG reste également un examen important dans la prise en charge des CIA surtout pour la recherche de troubles du rythme et de la conduction [16]. Dans notre série, on retrouvé essentiellement des signes en rapport avec le retentissement avec un axe droit et une hypertrophie cavitaire droite intéressant surtout l'oreillette droite. Il en était de même dans les autres travaux réalisés au Sénégal ainsi qu'au Maroc avec Rhizlaine [3, 17, 14]. Comme l'a montré notre travail, deux anomalies biologiques restent fréquentes, l'anémie et l'hyperleucocytose. La première souvent liée à un contexte de polycarence surtout martial, la seconde aux infections associées qui révèlent parfois la cardiopathie. Diop I et Wone R au Sénégal ont fait le même constat ainsi que Rhizlane au Maroc [3, 10, 14].

Sur le plan thérapeutique, chez 91% de nos patients, un traitement diurétique était prescrit; ceci témoigne du caractère retentissant sur la fonction cardiaque des CIA en milieu hospitalier nécessitant ce traitement. Les prescriptions de diurétiques étaient moindres dans la série de Bah [13] et Diop [10] avec environ 50% de cas. En Europe, les diurétiques restent encore moins prescrits du fait du diagnostic et de la prise en charge précoce. Cinq de nos patients soit 67% avaient bénéficié de fermeture par patch et 2 soit 28% de fermeture directe, trois ont été sous circulation extracorporelle (CEC). Dans l'étude de Rhizlaine [14] au Maroc tous les patients avaient bénéficié d'une chirurgie curative avec une fermeture par patch dans 91% des cas et une fermeture directe dans 9% et la moyenne des CEC était de 59,6%. Pour Marchall [19], la technique percutanée semblait plus sophistiquée et laissait moins de trace avec l'utilisation de ballon choisi après mesure de la taille de la CIA. Les résultats sont généralement excellents avec très peu de complications ou de décès cependant dans notre travail nous avons déploré deux cas soit 4% de décès en post opératoire. Parfois la chirurgie n'est pas proposée d'emblée car dans certains cas une fermeture spontanée est possible en règle dans la première année de vie et pour certain jusqu'à l'âge de 5 ans [18]. Une relation statistique significative a été notée avec un test du khi-deux inférieur à 0,05 entre l'âge et la chirurgie curative de même qu'entre l'apparition de signes d'insuffisance cardiaque et la cure chirurgicale. Ainsi on peut dire que dans certains cas plus l'enfant avance en âge, plus il apparait des signes d'insuffisance cardiaque et plus la chirurgie s'impose. Par contre la prise en charge chirurgicale n'est souvent pas réalisée chez les patients de jeunes âges dans notre contexte.

## Conclusion

Au total, la CIA est une cardiopathie congénitale fréquente en milieu hospitalier souvent de type ostium secundum, dont le pronostic est, à long terme, excellent et essentiellement conditionné par la précocité du diagnostic et de la prise en charge. La prise en charge médicale est essentiellement symptomatique et préventive pour l'endocardite mais l'indication opératoire requiert un peu plus d'expertise. Les complications au moment du diagnostic sont l'apanage des formes évoluées ou avec retentissement surtout chez le nourrisson, constituées essentiellement des infections pulmonaires et de l'insuffisance cardiaque.

### Etat des connaissances actuelles sur le sujet

- L'épidémiologie est très bien décrite en occident;
- L'embryologie est assez exhaustive;
- Les formes avec retentissement sont peu fréquentes.

### Contribution de notre étude à la connaissance

- Les circonstances du diagnostic;
- Les manifestations cliniques fréquentes orientant au diagnostic;
- Quelques aspects de la prise en charge locale.

## Conflits d’intérêts

Les auteurs ne déclarent aucun conflit d'intérêts.

## Remerciements

Ils sont addressés au Pr Mouhamadou Ndiaye du service de CTCV de Fann, Pr Amadou Lamine Fall du CHNEAR, Pr Maboury Diaw du service de cardiologie de l?HALD, Pr Ousmane Ndiaye, Chef de service de la pédiatrie, Dr Fatou Niang, Pr Ndéye Rama Diagne/Guéye.
